# Deciphering the Role of Trehalose in *Chroococcidiopsis* sp. 029’s High-Desiccation Resistance: Sequence Determination, Structural Modelling and Simulative Analysis of the 30S Ribosomal Subunit

**DOI:** 10.3390/molecules29153486

**Published:** 2024-07-25

**Authors:** Davide Pietrafesa, Alessandro Napoli, Federico Iacovelli, Alice Romeo, Fabio Giovanni Tucci, Daniela Billi, Mattia Falconi

**Affiliations:** Department of Biology, University of Rome Tor Vergata, Via della Ricerca Scientifica 1, 00133 Rome, Italy; davide.pietrafesa@uniroma2.it (D.P.); alessandro.napoli@uniroma2.it (A.N.); federico.iacovelli@uniroma2.it (F.I.); alice.romeo@uniroma2.it (A.R.); fabio.giovanni.tucci@uniroma2.it (F.G.T.); billi@uniroma2.it (D.B.)

**Keywords:** cyanobacteria, *Chroococcidiopsis* sp. 029, ribosomal 30S subunit, molecular dynamics simulations, desiccation resistance, molecular mechanism

## Abstract

Desert strains of the genus *Chroococcidiopsis* are among the most desiccation-resistant cyanobacteria capable of anhydrobiosis. The accumulation of two sugars, sucrose and trehalose, facilitates the entrance of anhydrobiotes into a reversible state of dormancy by stabilizing cellular components upon water removal. This study aimed to evaluate, at the atomistic level, the role of trehalose in desiccation resistance by using as a model system the 30S ribosomal subunit of the desert cyanobacterium *Chroococcidiopsis* sp. 029. Molecular dynamic simulations provided atomistic evidence regarding its protective role on the 30S molecular structure. Trehalose forms an enveloping shell around the ribosomal subunit and stabilizes the structures through a network of direct interactions. The simulation confirmed that trehalose actively interacts with the 30S ribosomal subunit and that, by replacing water molecules, it ensures ribosomal structural integrity during desiccation, thus enabling protein synthesis to be carried out upon rehydration.

## 1. Introduction

Water is essential for life; nevertheless, a few organisms found across different taxa, like bacteria, cyanobacteria, yeasts, lichens, nematodes, tardigrades, bdelloid rotifers, and resurrection plants, enter an ametabolic state upon water removal and resume normal activities upon rehydration, a phenomenon known as anhydrobiosis [1]. Upon desiccation, a wide array of damages is induced that is lethal to most of the organisms. Lipid membranes undergo a phase transition; carbohydrates, proteins, and nucleic acids experience Maillard reactions; and enzyme activities are inhibited, thus causing the production of reactive oxygen species [2]. A common feature of anhydrobiotes is that they contain a large amount of two non-reducing disaccharides, trehalose and sucrose, in the dry state [3]. These two sugars give rise to a sort of glassy matter that provides desiccation tolerance by weakening molecular diffusion and blocking chemical reactions, thus ensuring survival after prolonged air drying [3].

Among anhydrobiotic cyanobacteria, desert strains of the genus *Chroococcidiopsis* were extensively investigated under laboratory conditions, demonstrating the capability of air-dried cells to recover upon rehydration after several years of air-dried storage [4,5]. In particular, *Chroococcidiopsis* sp. 029 isolated from an endolithic community collected in the Negev Desert was reported to over-express genes for the biosynthesis of trehalose and sucrose that were accumulated in response to desiccation [6]. Moreover, a reduced copy number of 16S rRNA and a sub-set of mRNAs were still detectable in 7-year air-dried cells [7]. It was anticipated that the permanence in the dried cells of a stabilized translational apparatus (i.e., ribosome and ribosomal RNAs) along with a dormant transcriptome might be one the most intriguing aspects of *Chroococcidiopsis*’s reactivation after prolonged air drying [7]. Therefore, it is relevant to elucidate how non-reducing disaccharides can guarantee the integrity of anhydrobioses’ translational apparatus. Such knowledge will not only contribute to deciphering how this cyanobacterium can withstand life-limiting conditions in desert environments but will also contribute to developing cyanobacterial-based technologies to support future human outposts on the Moon or Mars [8,9]. Indeed, the resistance of *Chroococcidiopsis* sp. 029 is not exclusively linked to extreme conditions on Earth since it can cope with extra-terrestrial conditions, such as Mars-like conditions simulated on the ground or during space experiments performed outside the International Space Station [10,11].

Cyanobacteria can survive in different environments, including extreme ones such as glaciers, deserts, humid rocks, and hypersaline environments [2,3]. They can adapt to different environmental conditions and use effective protection mechanisms against various abiotic stresses. Some can withstand high levels of ultraviolet radiation or high light intensities, while others have a marked tolerance to water stress or high heat resistance [1,4,5].

In this paper, various computational tools, including metaSPades, a versatile metagenomic assembler [12], and PROKKA (https://github.com/tseemann/prokka, accessed on 19 July 2024), a command line software tool used to fully annotate a draft bacterial genome [13], were used to recover the sequences of 21 proteins and 16S ribosomal RNA from Illumina sequencing data, which constitute the 30S minor subunit of the cyanobacterium.

To generate a three-dimensional model for each protein, suitable templates were identified from these protein sequences using the SWISS-MODEL web server (https://swissmodel.expasy.org/, accessed on 19 July 2024) [14], and the 21 three-dimensional models were built using the Modeller program (https://salilab.org/modeller/, accessed on 19 July 2024) [15]. The structure of 16S ribosomal RNA was obtained using x3DNA software (https://x3dna.org/, accessed on 19 July 2024) [16], and the entire system was assembled using UCSF Chimera software (https://www.cgl.ucsf.edu/chimera/, accessed on 19 July 2024) [17]. Moreover, to elucidate *Chroococcidiopsis* sp. 029’s dehydration resistance, six 250 ns classical molecular dynamic (MD) simulations of the 30S subunit in the absence or presence of 0.6 M of trehalose molecules were carried out to evaluate, at the atomistic level, the potential role of trehalose regarding its resistance to desiccation. In fact, previous studies demonstrated the ability of molecular dynamic simulation in modelling protein–threalose interactions [18,19]. The modelled 30S ribosomal subunit provided an accurate atomistic description of the interactions between the disaccharide and the ribosome, confirming the reliability of the obtained model. The direct interactions between the trehalose disaccharides and the *Chroococcidiopsis* 30S ribosomal subunit make the entire 30S structure very stable, placing it in a quiescent state and confirming the observed mechanism of protein synthesis weakening.

## 2. Results

### 2.1. Sequence Identification

The 21 protein sequences and the sequence encoding the 16S rRNA molecule, which make up the 30S ribosome subunit of the cyanobacterium *Chroococcidiopsis* sp. 029, were identified using the programs FASTQC (https://www.bioinformatics.babraham.ac.uk/projects/fastqc//, accessed on 19 July 2024) [20], TRIMMOMATIC (http://www.usadellab.org/cms/?page=trimmomatic, accessed on 19 July 2024) [21], metaSPAdes (https://bio.tools/metaspades, accessed on 19 July 2024) [12], and PROKKA (https://github.com/tseemann/prokka, accessed on 19 July 2024) [13], as indicated in the Computational Methods section.

### 2.2. Models Building

**Figure 1:**
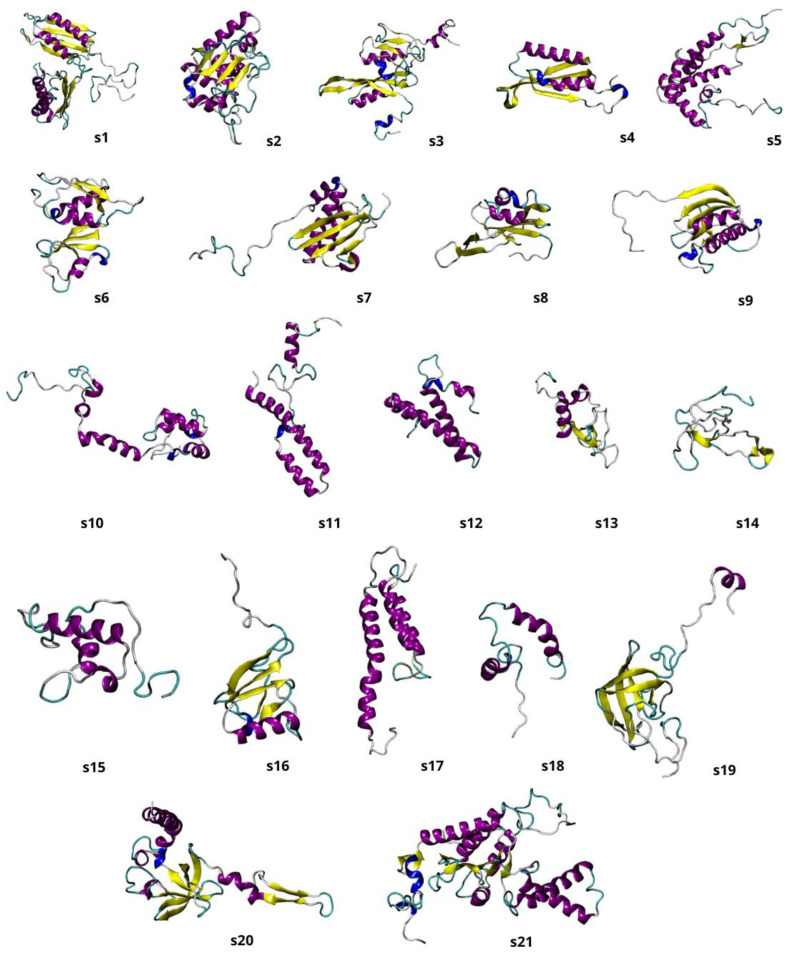
Graphical representation of the 3D models of the 21 30S ribosome proteins, indicated as s1–s21, obtained with homology modelling. The different colors represent diverse secondary structures: in purple, the α-helices; in yellow, the β-sheets; and in light grey, the loops.

**Figure 2:**
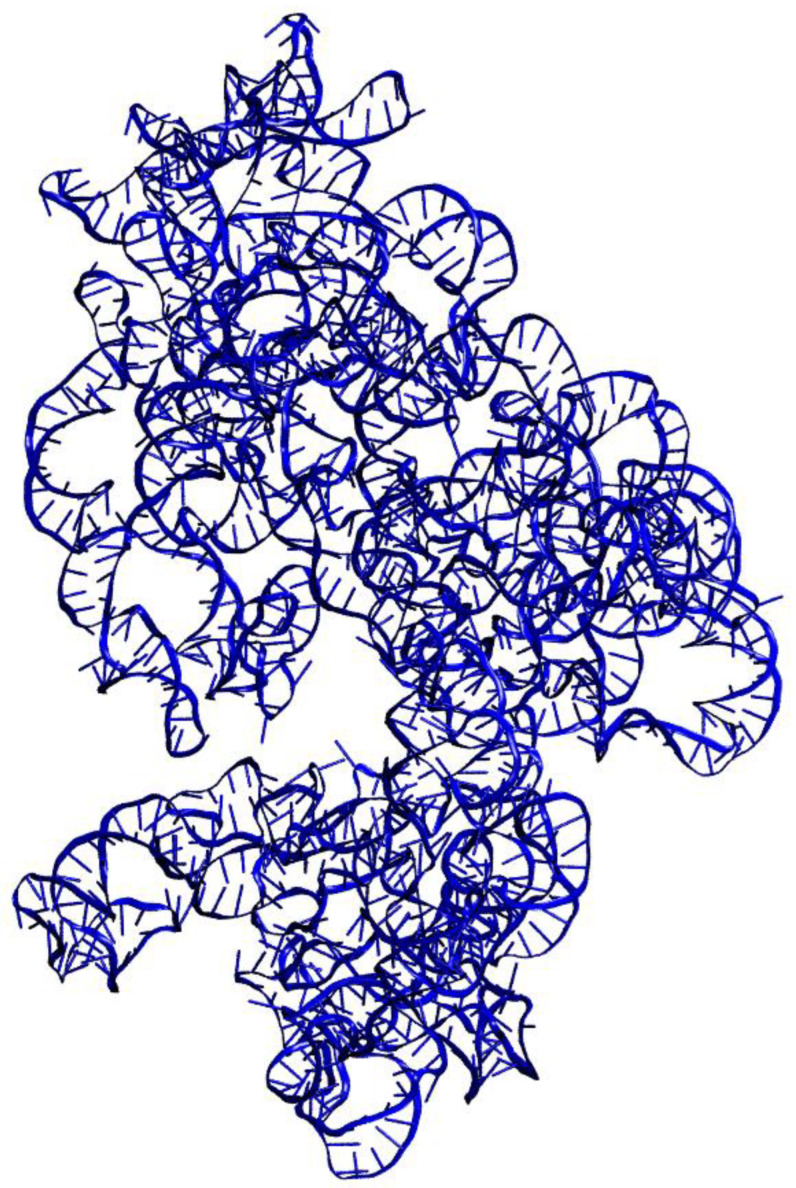
Three-dimensional model of the 16S rRNA of the cyanobacterium *Chroococcidiopsis* sp. 029.

**Figure 3:**
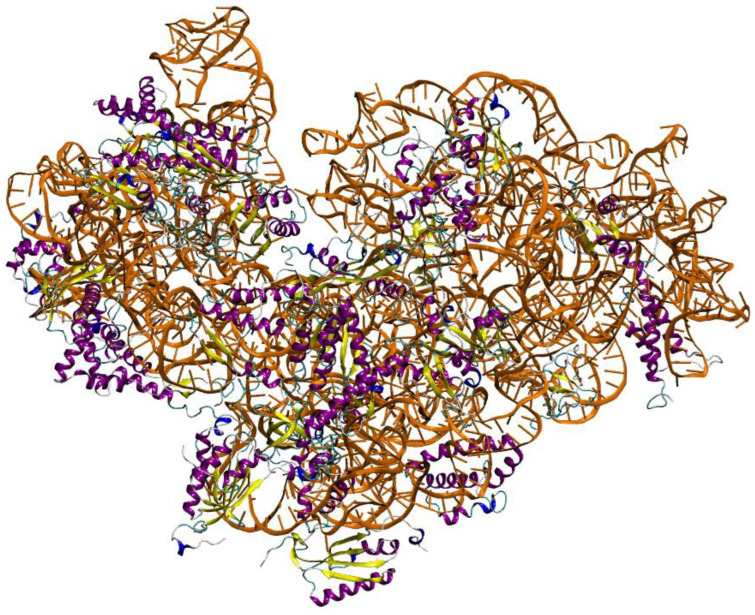
Model of 30S ribosome subunit of *Chroococcidiopsis* sp. 029 after complete assemblage. The 16S rRNA is depicted in orange, while the 21 proteins are colored by secondary structure.

For each of the 21 target protein sequences, five models were generated using the Modeller program [15]. Each protein’s best three-dimensional (3D) model was selected based on DOPE score values. Discrete Optimized Protein Energy (DOPE) represents a statistical potential used to evaluate the quality of a homology model: the lower the DOPE score, the better the model [22]. Figure 1 shows the 3D representations of each of the 21 generated structures; Figure 2 shows the 3D model of the 16S rRNA molecule of *Chroococcidiopsis* sp. 029 obtained with x3DNA [16]; and Figure 3 represents the entire 30S ribosome assembled model.

### 2.3. RMSD, RMSF, Gyration Radius, and Secondary Structure Analyses

**Figure 4:**
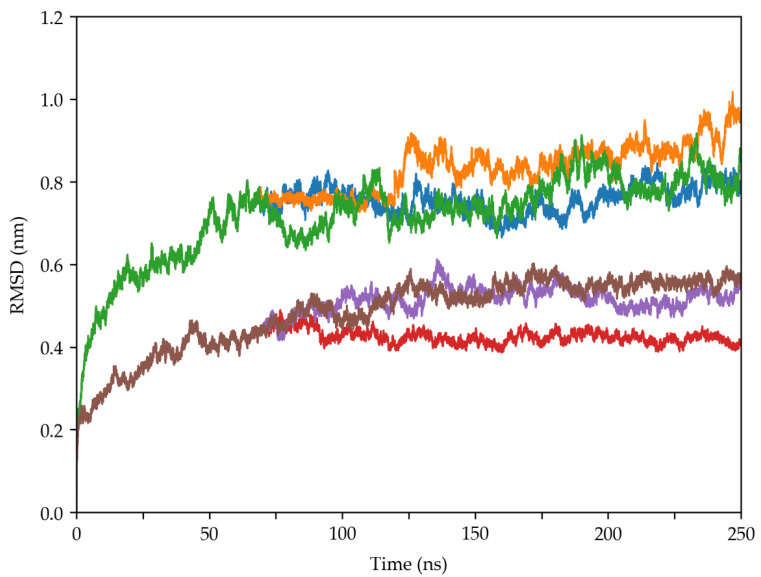
Time evolution of RMSD values of the 16S RNA molecules in the two systems as a function of time. The blue, orange, and green lines indicate the three MD replicas simulated without trehalose, while the red, violet, and brown lines indicate those simulated in the presence of trehalose molecules.

**Figure 5:**
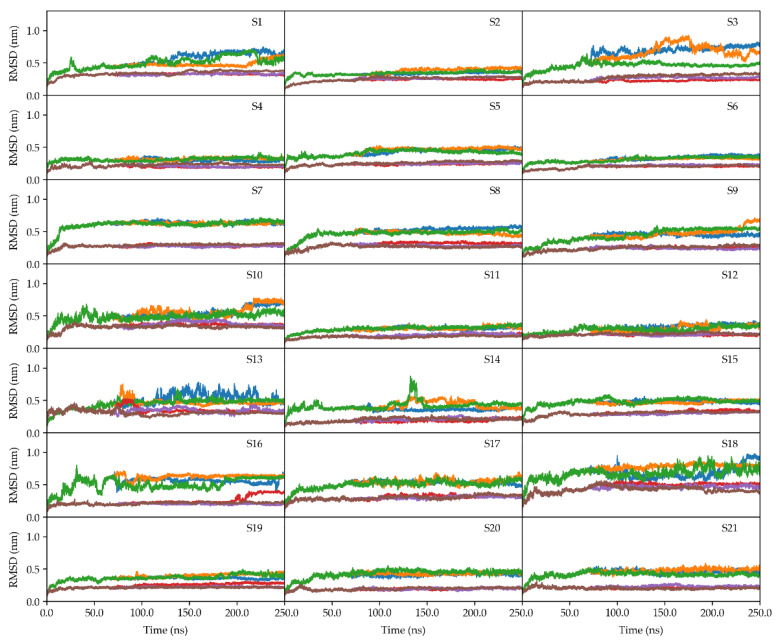
RMSD values as a function of time for the 21 proteins of the 30S subunit in the two systems, indicated as S1–S21, as a function of time. The blue, orange, and green lines indicate the three MD replicas simulated without trehalose, while the red, violet, and brown lines indicate those simulated in the presence of trehalose molecules.

To assess the overall stability and the impact of the sugar on the systems of MD simulations, the RMSDs of the 16S rRNA and 21 protein subunits were monitored for three simulation replicas with and without trehalose (Figure 4 and Figure 5).

Both systems reached convergence after 100 ns in all simulation replicas. Notably, the systems with trehalose demonstrated consistently lower RMSD values across both 16S RNA and protein subunits. These results highlight trehalose’s potential as a critical protective agent in desiccation resistance. As observed in desiccation-tolerant organisms, trehalose directly interacts with nucleic acids, proteins, and membranes, preserving the native structure of these molecules [3].

Root Mean Square Fluctuation (RMSF) was calculated for the RNA and for each protein in all systems’ replicas to assess residue-level flexibility throughout the simulations (Figure S1 and Figure S2A,B, respectively, Supplementary Materials). As expected, unstructured terminal regions exhibited higher RMSF values than the residues that were involved in secondary structures. Notably, the system with trehalose demonstrated a slightly lower average residue flexibility across the RNA and proteins in all the replicas, implying a stabilizing effect on the 30S ribosomal subunit.

Secondary structures were analysed throughout the simulations to further investigate the impact of trehalose on protein structure (Table S4, Supplementary Materials). Notably, the presence of trehalose slightly altered the secondary structure content of almost all proteins compared to the systems simulated without trehalose. This observation further supports the stabilizing role of trehalose during desiccation by maintaining protein structure.

Radius of gyration (RG) analysis was used to assess the compactness of the macromolecular structures (Figure S3, Supplementary Materials). RG calculations were performed on the 30S proteins and 16S rRNA for the three simulation replicas of the two systems. As can be observed, the 30s subunits simulated in the presence of trehalose molecules are more compact with respect to those simulated in the absence of sugar, even if they are both characterized by high stability throughout the simulation.

### 2.4. Principal Components Analysis

Principal component analysis (PCA) is a powerful tool for analysing MD simulations. It identifies a biomolecule’s dominant, collective motions by transforming complex trajectory data into a reduced set of essential coordinates (principal components).

**Figure 6:**
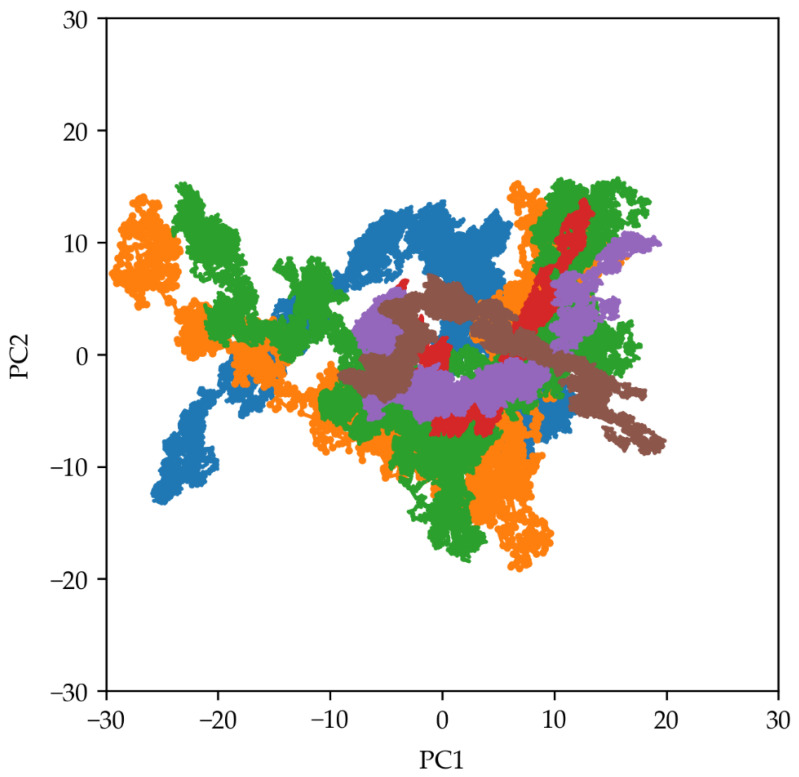
Two-dimensional plot showing the projections of the first (PC1) and second (PC2) principal components obtained from the PCA analyses. The blue, orange, and green lines indicate the three MD replicas simulated without trehalose, while the red, violet, and brown lines indicate those simulated in the presence of trehalose molecules.

PCA was performed on the 2747 Cα atoms to identify the main collective motions within the 21 proteins of the 30S ribosomal subunit. Starting from the diagonalization of the covariance matrices, the two-dimensional (2D) projections of the first and second principal components (PC1 and PC2, respectively) were plotted for all the simulation replicas of the two systems (Figure 6). The 2D projections for the systems simulated without trehalose exhibited a wider distribution than those with trehalose. This observation suggests that trehalose confines the conformational space the ribosome explores, enhancing its structural stability.

**Figure 7:**
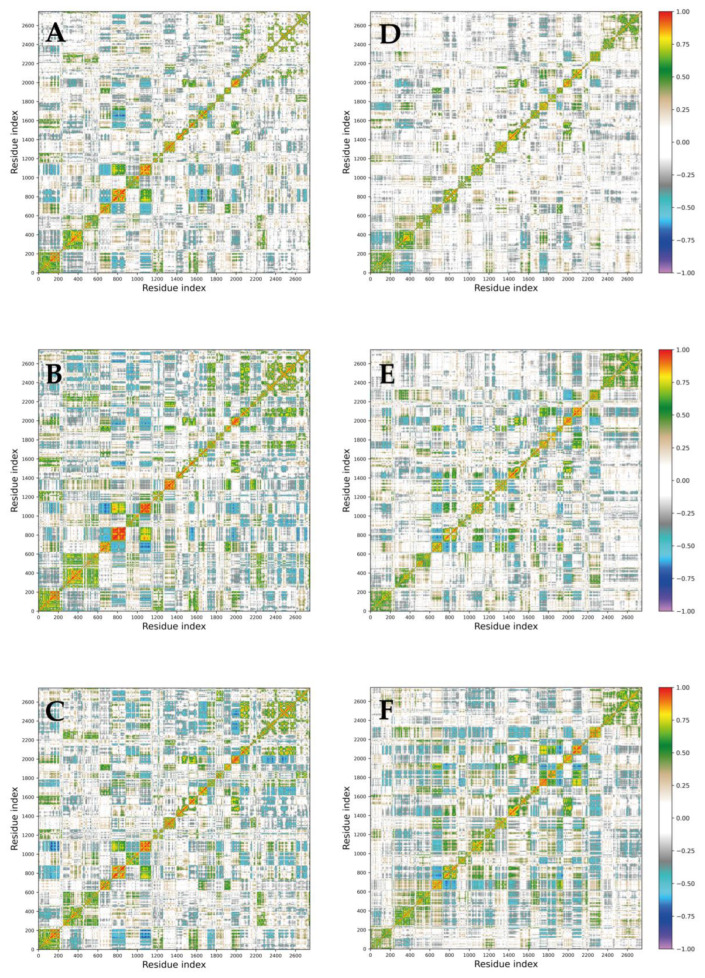
DCCMs obtained from the covariance matrices of the 30S subunits. The (**A**–**C**) panels represent the DCCM calculated on Cα atoms for the three replicas simulated without sugar molecules, while the (**D**–**F**) panels represent the DCCMs calculated for the replicas simulated with trehalose. Colour coding is reported in the legend.

Dynamic cross-correlation maps (DCCMs) were generated to quantify the degree of correlation between the movements of different atoms within the 30S ribosomal subunit and investigate the impact of trehalose on structural dynamics. These maps were calculated using the atomic fluctuations of Cα atoms from the 21 proteins in each MD trajectory (Figure 7). Positive values in the DCCMs indicate correlated motion (residues moving in the same direction), while negative values indicate anti-correlated motion (residues moving in opposite directions). A comparison of DCCMs revealed a subtle decrease in both correlated and anti-correlated motions within the trehalose systems (Figure 7D–F) with respect to the ones simulated in the absence of sugar (Figure 7A–C). This observation indicates that trehalose stabilizes the 30S ribosomal structure, potentially restricting its internal motions.

### 2.5. Hydrogen Bond Analysis

Hydrogen bonds were analysed to investigate trehalose’s potential to form direct interactions with the 30S structure, which should prevent denaturation during desiccation [3].

**Figure 8:**
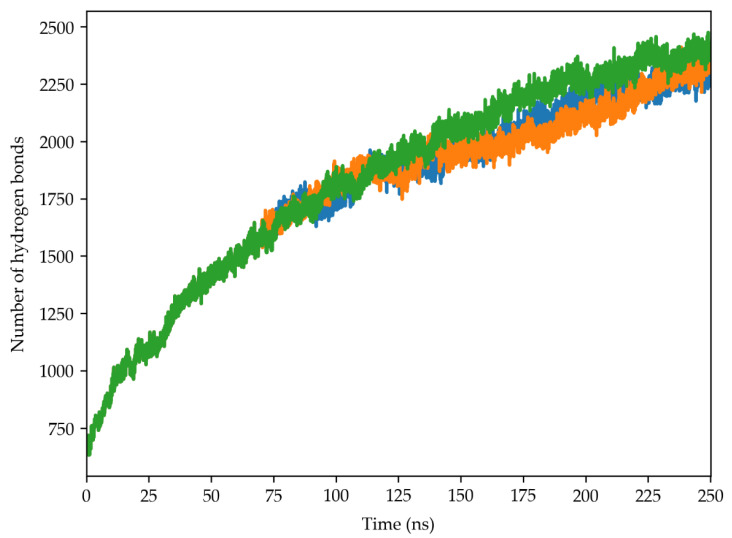
Time evolution of the hydrogen bond number established between the trehalose molecules and the 30S ribosome structure of the cyanobacterium *Chroococcidiopsis* sp. 029. The blue, orange and green lines indicates the three MD simulation replicas.

This analysis revealed a significant increase in hydrogen bonds between trehalose and the 30S ribosomal subunit throughout the three simulation replicas (Figure 8). The number of hydrogen bonds rose steadily from an initial value of approximately 650 to around 2300 at the simulation’s end. This finding shows trehalose’s high propensity to directly interact with the proteins and rRNA within this complex structure.

### 2.6. Radial Distribution Function

**Figure 9:**
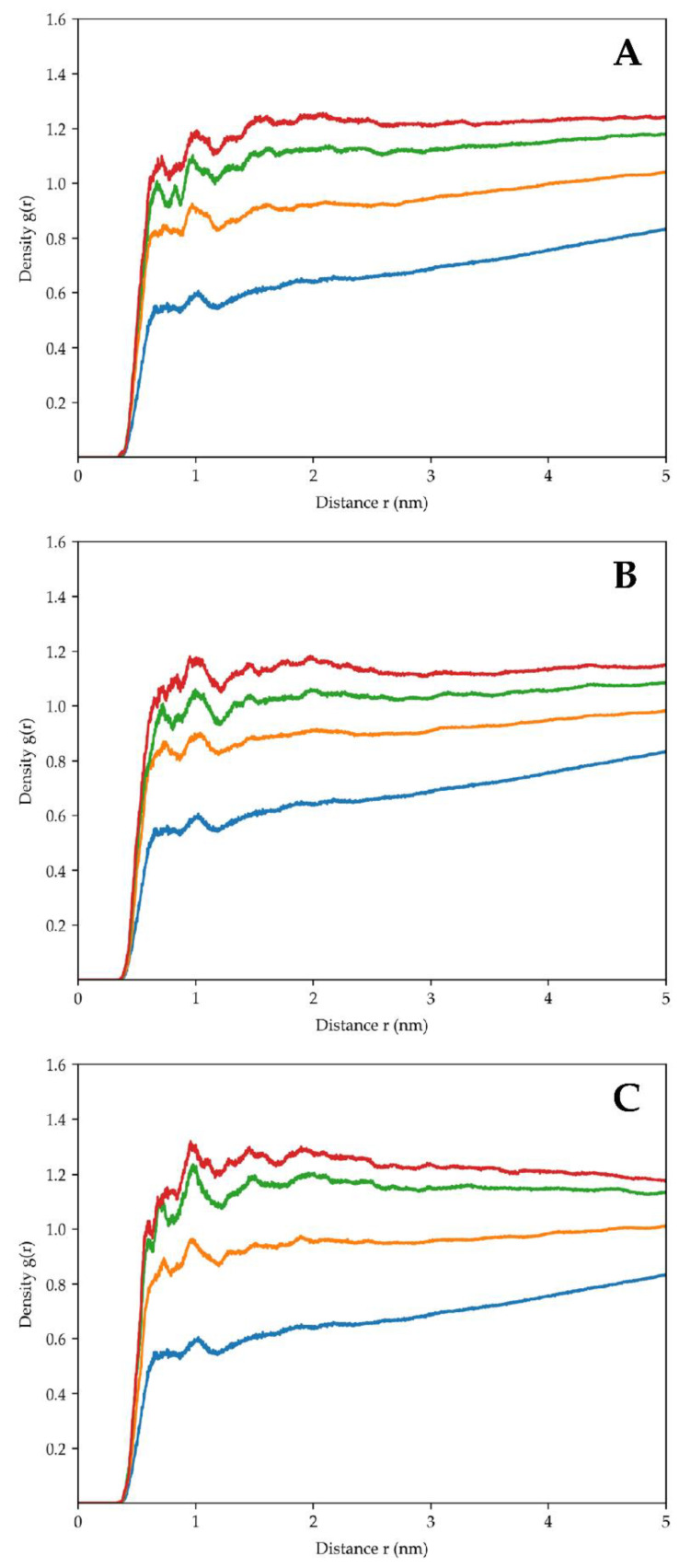
The radial distribution function of trehalose indicates that the density of the sugar, concerning the ribosome surface, increases over time. Each function was calculated in a different and progressive simulation time window. The values increase from the blue (50–100 ns) to the red (200–250 ns) line. The (**A**–**C**) panels depict trehalose’s behaviour in the three simulation replicas.

The radial distribution function (RDF) of trehalose around the ribosomal surface, calculated at four 50 ns time windows, was evaluated throughout the three simulation replicas. To consider the structural equilibration of trehalose’s system, the first interval (i.e., 0 to 50 ns) was not considered in the analysis. Figure 9A–C demonstrates a progressive increase in trehalose density around the ribosomal subunit, as evidenced by the rising peaks. The density curves steadily rise within each interval, indicating that trehalose molecules progressively accumulate around the structure, forming a protective envelope around the 30S subunits and supporting the hypothesis that trehalose exerts a stabilizing effect on the cyanobacterial ribosome.

## 3. Discussion

The MD simulations presented in this research revealed that the presence of trehalose enhances the stability of the 30S ribosomal subunit. This observation is supported by RMSD (Figure 4 and Figure 5), RMSF (Figures S1 and S2, Supplementary Materials), and secondary structure analyses (Table S4, Supplementary Materials), which consistently showed lower fluctuations and better preservation of structural integrity in the presence of trehalose.

These findings corroborate previous studies that suggested that trehalose is a stabilizing agent, protecting biomolecules from denaturation and preserving their native structure during desiccation or other stress conditions [2,3,6,18,19].

The analysis of hydrogen bonds (Figure 8) and RDF (Figure 9) provided insights into the molecular interactions between trehalose and ribosomal subunit components. The significant increase in hydrogen bonds over the simulation suggests that trehalose forms direct and extensive contacts with the ribosomal proteins and RNA, reinforcing the stability of the complex. Moreover, the accumulation of trehalose around the ribosomal surface, as indicated by RDF analysis, suggests the progressive formation of a protective envelope that shields the ribosome from environmental stressors.

PCAs and DCCMs (Figure 6 and Figure 7, respectively) elucidated the collective motions within the ribosomal subunit and revealed a stabilizing effect of trehalose on structural dynamics. The narrower distribution of principal components and reduced correlated and anti-correlated motions in the presence of trehalose indicated more confined conformational space sampling and decreased structural fluctuations. This suggests that trehalose stabilizes the ribosomal subunit and influences its dynamic behaviour, potentially preserving its functional properties under stress conditions.

Understanding the molecular mechanisms underlying stress tolerance in organisms like cyanobacteria can have significant implications for biotechnological applications. By elucidating the protective role of trehalose in maintaining the integrity of essential cellular structures, such as the ribosome, this research opens avenues for developing strategies to enhance stress tolerance in various organisms, including crops, biofuel producers, and industrial microorganisms. By harnessing the protective properties of trehalose, it may be possible to engineer organisms with improved resilience to environmental stresses, ultimately enhancing productivity and sustainability in agriculture, biotechnology, and other fields.

In conclusion, the findings presented in this manuscript contribute to understanding the molecular basis of desiccation tolerance and highlight the importance of trehalose as a protective agent in maintaining the structural integrity and functional stability of essential cellular components, such as the ribosome. This research advances our knowledge of fundamental biological processes and suggests potential practical applications in biotechnology and environmental science.

## 4. Materials and Methods

### 4.1. Computational Methods

#### 4.1.1. Identification of Genes Encoding the Elements of the 30S Ribosomal Subunit

Several computational tools were employed to identify genes encoding the 21 ribosomal proteins and 16S rRNA of a cyanobacterium’s 30S ribosomal subunit from Illumina sequencing data (Supplementary Materials, Tables S1 and S2). Initial sequencing quality was assessed using FASTQC (https://www.bioinformatics.babraham.ac.uk/projects/fastqc//, accessed on 19 July 2024) [20], followed by trimming or removing low-quality reads using TRIMMOMATIC (http://www.usadellab.org/cms/?page=trimmomatic, accessed on 19 July 2024) [21]. De novo genome assembly was performed using the metaSPAdes assembler (https://bio.tools/metaspades, accessed on 19 July 2024) [12], and gene annotation was conducted with PROKKA (https://github.com/tseemann/prokka, accessed on 19 July 2024) [13]. Finally, BLAST (https://blast.ncbi.nlm.nih.gov/Blast.cgi, accessed on 19 July 2024) [23] was employed against a curated database of *Chroococcidiopsis* protein sequences retrieved via UniProt (https://www.uniprot.org/, accessed on 19 July 2024) [24] to identify the genes of interest precisely.

#### 4.1.2. Molecular Modelling of the 21 Proteins and the 16S rRNA Molecule

Homology modelling, implemented through the Modeller program [15], was used to build three-dimensional models of the 21 ribosomal proteins. The SWISS-MODEL web server [14] facilitated the identification of suitable templates for each protein (Table S3, Supplementary Materials). The 16S rRNA structure was modelled using x3DNA software [16], using the 16S rRNA structure from the *Spinacia oleracea* chloroplast (PDB ID: 6ERI) as a template. Finally, the complete ribosomal subunit model was assembled using the Chimera program [17].

#### 4.1.3. Molecular Dynamics Simulations

Three classical 250 ns MD simulation replicas were carried out for the system in presence and absence of trehalose molecules. The first three modelled the *Chroococcidiopsis* sp. 029 30S subunit in a simple water box, while the remaining ones also included 4000 trehalose molecules (0.6 M concentration) surrounding the complex, introduced using the Packmol program (https://m3g.github.io/packmol/, accessed on 19 July 2024) [25]. The final systems accounted for 1,113,323 and 1,614,035 atoms, respectively. Given the size, 250 ns were considered a compromise to obtain meaningful data within reasonable timeframes. System preparation was conducted using the tLeap module of the AMBER22 suite (https://ambermd.org/, accessed on 19 July 2024) [26]. Parametrization employed the OL3 force field for the 16S rRNA [27], the ff19SB force field for the 30S proteins [28], and the Glycam06 force field for trehalose [29]. Systems were solvated in a cubic box of TIP3P water molecules [30] and neutralised with Na^+^ ions.

Energy minimization (500 steps steepest descent, 2000 steps conjugated gradient) removed unfavourable interactions. Initial thermalization (300 K) employed the Langevin thermostat [31] with gradually reduced constraints. Systems were then simulated in an NPT ensemble (1.0 atm pressure, Berendsen barostat) for 1.0 ns [32]. Production runs were performed for both systems using the AMBER22 pmemd.cuda module, saving coordinates every 1000 steps. Bonds involving hydrogen atoms were constrained with the SHAKE algorithm [33], long-range interactions were calculated via the PME method [34], and a short-range interaction cut-off of 8.0 Å was applied. Simulations were executed on two GeForce RTX 4080 GPUs (https://www.nvidia.com/, accessed on 19 July 2024).

#### 4.1.4. Simulation Analysis

The trajectories were analysed with modules in the GROMACS 2023 suite (https://www.gromacs.org/, accessed on 19 July 2024) [35]. The first 50 ns were not considered for the analyses that represented a thermalization phase for both systems. The RMSDs, RMSFs, and RG were computed by using rms, rmsf and gyrate modules, respectively. PCA of covariance matrices of the atomic fluctuations were calculated on the Cα atoms of the 30S subunit in the two systems [36] using the covar and anaeig modules. An in-house Python script obtained the DCCMs.

The Hbonds plugin of VMD 1.9.3 (https://www.ks.uiuc.edu/Research/vmd/, accessed on 19 July 2024) [37] was used to analyse hydrogen bonds between trehalose and the 30S subunit. A hydrogen bond was assumed to exist if the donor-to-acceptor distance was shorter than 0.30 nm and the hydrogen-donor-acceptor angle was lower than 30°.

The RDF of trehalose was calculated as a function of distance from the surface of the ribosome subunit using the rdf module of the GROMACS 2023 suite, applying the standard tool normalization parameters, which normalizes the rdf value by the average number of molecular positions [35]. Images were rendered using VMD (https://www.ks.uiuc.edu/Research/vmd/, accessed on 19 July 2024) [37] or Chimera software (https://www.cgl.ucsf.edu/chimera/, accessed on 19 July 2024) [17].

## 5. Conclusions

The research presented in this manuscript delves into the computational methods, molecular modelling, and MD simulations to explore the structural dynamics and stability of the cyanobacterium *Chroococcidiopsis* sp. 029’s 30S ribosomal subunit. By employing a comprehensive computational approach, this study aimed to elucidate the intricate details of this essential cellular structure and its response to environmental stressors, mainly focusing on the protective role of trehalose.

The genes encoding the components of the 30S ribosomal subunit were obtained from Illumina sequencing data, followed by molecular modelling of the ribosomal proteins and the 16S rRNA molecule. Subsequently, two 250 ns MD simulations were performed to investigate the dynamics of the ribosomal subunit, with and without trehalose, a known protective agent against desiccation.

The presented findings shed light on the structural dynamics and stability of the cyanobacterial ribosomal subunit, emphasizing the crucial role of trehalose in maintaining its integrity under environmental stresses. This research contributes to the understanding of cellular adaptations to challenging conditions and may have implications for biotechnological applications to enhance stress tolerance in various organisms.

## Data Availability

The data presented in this study are available on request.

## Supplementary Materials

The following supporting information can be downloaded at: https://www.mdpi.com/article/10.3390/molecules29153486/s1, Table S1. Sequences of the 21 proteins belonging to the 30S subunit of the cyanobacterium *Chroococcidiopsis* sp. 029. The first column shows the proteins names, canonically numbered from 1 to 21; the second column shows the corresponding amino acid sequence. Table S2. Nucleotide sequence of the 16S rRNA from *Chroococcidiopsis* sp. 029 identified in the assembly. Table S3. List of templates selected on SWISS-MODEL for comparative modeling. The first column shows the name of the protein, in the second the template chosen with the corresponding PDB entry; the third column shows the number of residues of the template sequence and the last column shows the organism to which the selected template belongs. Table S4. Average percentage of secondary structures for the 21 proteins of 30S ribosomal subunit. For each protein, the first row is related to the system simulated without trehalose, while the second row is related to the system simulated with trehalose. Figure S1. (A) RMSF calculated for the C2’ atoms of the RNA in the systems without trehalose and (B) for the system with trehalose. The blue, orange and green colours identify the three different simulation replicas. Figure S2. (A) RMSF calculated for each of the 21 proteins in the three system replicas simulated without trehalose and (B) for three system replicas simulated in presence of trehalose). In order, from left to right, the protein chains are shown from S1 to S21. The mean and the standard deviation of each curve is reported inside the plots. The blue, orange and green colours identify the three different simulation replicas. Figure S3. Gyration radius (RG): blue, orange and green lines indicate the RG values calculated for the three replicas in the absence of trehalose, while red, violet and brown lines the values calculated for the replicas simulated in presence of trehalose.
