# Supplementary material for: Deciphering the Role of Trehalose in Chroococcidiopsis sp. 029’s High-Desiccation Resistance: Sequence Determination, Structural Modelling and Simulative Analysis of the 30S Ribosomal Subunit
Source: Molecules. 2024 Jul 25;29(15):3486. doi: 10.3390/molecules29153486 (PMC11314286; doi:10.3390/molecules29153486)
Supplement: Supplementary file 1 [file molecules-29-03486-s001.zip › molecules-3036118-supplementary.pdf]

molecules

Deciphering the Role of Trehalose in *Chroococcidiopsis* sp. 029’s High-Desiccation Tolerance Resistance: Sequence Determination, Structural Modelling and Simulative Analysis of the 30S Ribosomal Subunit

Davide Pietrafesa, Alessandro Napoli, Federico Iacovelli, Alice Romeo, Fabio Giovanni Tucci, Daniela Billi and Mattia Falconi *

Department of Biology, University of Rome Tor Vergata, Via della Ricerca Scientifica 1, 00133 Rome, Italy; davide.pietrafesa@uniroma2.it (D.P.); alessandro.napoli@uniroma2.it (A.N.); federico.iacovelli@uniroma2.it (F.I.); alice.romeo@uniroma2.it (A.R.); fabio.giovanni.tucci@uniroma2.it (F.G.T.); billi@uniroma2.it (D.B.)

* Correspondence: falconi@uniroma2.it

Table S1. Sequences of the 21 proteins belonging to the 30S subunit of the cyanobacterium *Chroococcidiopsis* sp. 029. The first column shows the proteins names, canonically numbered from 1 to 21; the second column shows the corresponding amino acid sequence.

Sequence	30S Ribosomal Protein
MVNQKLTATEIGFTHDDFAALLDKYDYHFSPGDIVAGTVFSIEPRGALI<br>DIGAKTAAYIPIQEMSINRVDAPEEVLQSNETREFFILTDENEDGQLTLS<br>IRRIEYMRAWERVRQLQTEDATVRSIVFATNRGGALVRIEGLRGFIPGS<br>HISTRKPKEDLVGEELPLKFLEVDEDRNRLVLSHRRALVERKMRLEVG<br>EVVIGTVRGIKPYGAFIDIGGVSGLLHISEISHDHIDTPHSVFNVNDEVK<br>VMIIDLDAERGRISLSTKQLEPEPGDMIKDRQLVYDKAEEMAVRYREQ<br>MLAKQQGITVAAPESEASAESEEIPSAVEEYIPSAVEEELPVAVEEELPA<br>AVEE	S1
MPVVSLAQMMESGVHFGHQTRRWNPKMSPYIYTARNGVHIIDLVQT<br>AQLMEEAYNHIRSEAEQGKKFLFVGTKRQAAGIVAQEAARCGSHYIN<br>QRWLGGMLTNWATIKTRVERLKDLERREESGALDLLPKKEASVLRRE<br>MEKLQKYLGGIKTMRKVPDAVVIVDQRREYNAVQECQKLGVEIVSML<br>DTNCDPDVVDIPIPANDDAIRSIKLIVGKLADAIYEGRHGQLDAEEDY<br>EDYEGAEEDFDDDESESEYTDSLIPDQEEEETQE	S2
MGQKIHPIGFRLGITKDHHSRWFAEPARYPEILQEDYKLRQYIEQKLG<br>RLAANNAGISDVRIERKADQIELEVRTARPGVVVGRGGAGIESLRTGL<br>QELLGGNRQIRINVVEVQQVDADAYLIAEYIAQQLERRVSFRRVVRQA<br>IQRAQRAGIQGIRIQVSGRLNGAEIARTEWTREGRVPLHTLRADIDYAY<br>CTAKTVYGILGVKVWIFKGEIIPGQEQQPTPGAAQPRRRQQQRRRQQF<br>EDRSNEG	S3
MSRYRGPRLRIVRRLGDLPGLTRKSARRAYPPGQHGQARKKRSEYAIR<br>LEEKQKLRFNYGLTEKQMLRYVRRARRVTGSTGQVLLQLLEMRLDNT<br>VFRLGMAPTIPAARQLVSHGHVTVNGRVVNIPSYQCRPGEAIAVANR<br>EQSRKLVETNLQYPGLANLPSHLEFDKNKLEGRVNGLVEREWIALQV<br>NELLVVEYYSRQA	S4
MATGRRKSNRVKEKETTWQERVIQIRRVSKVVKGGKKLSFRAVVAV<br>GNERGQVGIGVGKAGDVIGAVKKGVADGKKHLVEIPLTKSNSIPHPI<br>NGVGGGAKVMMRPASPGTGVIAGGAVRTVLELAGVRNILAKQLGS<br>NNPLNNARAAVNALSTLRTFSEVADERGVPIENLYA	S5
MPSAYETMYILRPDLGEEQIEQAIAKYENFLKDQGATNLQIQLRGKRR<br>LAYEIGRQREGVYVQMNYEAPGTAIAPMERAMRLSEEVIRYLTLKSEE<br>PTATPEAAAV	S6
MSRRTVAQKRPVPPDSVYNSRLISMMMRRVMHSGKKSIAAGIIYDAL<br>KTIEERTGAEPLETFERAVRNATPLVEVKARRVGGATYQVPMEVRAE<br>RGTALALRWLIQFSRQRPGRSMASKLANELMDAANETGNAIRKREE<br>THRMAEANKAFAHYRY	S7
MAANDTIADMLTRIRNANMARHQNTQIPATKMTRSIAKVLQDEGFI<br>AEIEEVEEGVKRNLVISLKYKGKNRQPLITALKRVSKPGLRVYSNRKEL<br>PRVLGGIGIAIISTSHGIMTDREARRQGLGGEVLCYIW	S8
MQALDTTNGRAMYWGTGRRKSSVARVRLVPGNGQLIVNGKPGDLY<br>FQFNANYLSVAKAPLETLGLENEYDILVNAHGGGLTGQSDSIRLGVAR<br>ALCQLDPSNRSPLKIEGYLTRDPRAKERKKYGLHKARKAPQFSKR	S9

MATLQQQKIRIRLQAFDRRLLDTSCEKIVDTANRTNATAIGPIPLPTKRRIYCLLRSPHVDKDSREHFETRTHRRIIDIYQPSSKTIDALMKLDLPSGVDIEVKL	S10
MARQPTKKSGTKKQKRNVPNGIAYIQSTFNNSIITIADQNGDVISWASAGSSGFKGAKKGTPFAAQTAAESAARRAVDQGMRQLEVMVSGPGAGRETAIRALQGAGLEITLIRDTTPIPHNGCRPPKRRRV	S11
MPTIQQLIRDERQIASTKTKSPALKQCPQRRGVCTRVYTTTPKKPNSALRKVARVRLTSGFEVTAYIPGIGHNLQEHSVVMIRGGRVKDLPGVRYHIIRGTLDTAGVKDRRQGRSKYGTKRPKST	S12
MARIAGVDLPRDKRVEIGLTYIYGIGLTRSKEILAAAGVNPDTRVKELSDADVAALRGEVEANYQVEGDLRRLEAMNIKRLIDIGSYRGRRHRMGLPVRGQRTRTNARTRRGRRQTVAGKKKAPSSK	S13
MAKKSMIEREKKRKKIVVKYAEKRQALIEQFENAASQTEKLEIHRQIQQLPRNSSRTRVNNRCWVTGRPRGVYRDFGLSRNVMREWAHEGLLPGVVKSSW	S14
MALTQQRKQELISDYQLHDTDTGSSEVQIAMLTERINRLSEHLRGNQKDHSSRRGLLKLIGQRKRLLSYIQQEDRQRYQALIARLGIRG	S15
MIKLRLKRYGKKREASYRIVVINSRDRRDGRPLEELGFYNPRTDETKLDVPGLVKRLQQGAQPTDTVRRILEKANVFEQVSTSATQ	S16
MAVKERVGLVVSDKMEKTVVVAVENRAPHPKYGKIVVRTQRYKAHDEENKCKVGDRVRIQETRPLSRTKRWMVKDILSSATTS	S17
MSYYRRRLSPIKPQEPIDYKDVDLLRKFVTERGKILPRRITGLTAKQQRDLTLAIKRSRIVALLPFINQEG	S18
MGRSLKKGPFVADSLLRKIEALNARGEKQVIKTWSRASTILPQMVGHTIAVHNGRQHVPIYLSEQMVGHKLGEFAPTRTFRGHAKSDKKAGR	S19
MANTKSAIKRVKIAERNRLRNKSYKSAVKTLMKKYLSAVETYTANPTPELQQEVQQHMAAAYSKIDKAVKTGVLHRNNGARKKSRLAKKLKQFETATSTAE	S20
MTQVIPGENEGIESALRRFKREVSKAGIFPDMRKHRHFETPIEKRKRKAVAKHKQRKRSSRR	S21

Table S2. Nucleotide sequence of the 16S rRNA from *Chroococcidiopsis* sp. 029 identified in the assembly.

Sequence	Name
UUCGGACACAGUGGCGGACGGGUGAGUAACGCGUGAGAA<br>UCUGGCCUUGGGUUCGGGACAACCACGGGAAACGGUGGC<br>UAAUACCGGAUGUGCAGAGAUGCAAAAGAUUAAUUGCCU<br>GAGGAUGAGCUCGCGUCUGAUUAGCUAGUUGGUGGGGUA<br>AAGGCCUACCAAGGCGGCGAUCAGUAGCUGGUCUGAGAG<br>GACGAUCAGCCACACUGGGACUGAGACACGGCCCAGACU<br>CCUACGGGAGGCAGCAGUGGGGAAUUUUCCGCAAUGGGC<br>GAAAGCCUGACGGAGCAAUACCGCGUGAGGGAGGAAGGC<br>UCUUGGGUUGUAAACCUCUUUUCUCAGGGAAGAAUACAA<br>UGACGGUACCUGAGGAAUAAGCAUCGGCUAACUCCGUGC<br>CAGCAGCCGCGGUAAUACGGAGGAUGCAAGCGUUAUCCG<br>GAAUGAUUGGGCGUAAAGCGUCCGCAGGUGGCAUUGCAA<br>GUCUGCUGUCAAAGCUCAGGGCUUAACCCUGAAAAGGCA<br>GUGGAAACUGAAAUGCUGGAGUGCGGUAGGGGUAGAGG<br>GAAUUCCCGGUGUAGCGGUGAAAUGCGUAGAGAUCGGGA<br>AGAACACCAGUGGCGAAAGCGCUCUACUAGGCCGCAACU<br>GACACUCAUGGACGAAAGCUAGGGGAGCGAAUGGGAUUA<br>GAUACCCCAGUAGUCCUAGCUGUAAACGAUGGAUACUAG<br>GCGUUGAGAGUAUCGACCCUCGCAGUGCCGUAGCUAACG<br>CGUUAAGUAUCCCGCCUGGGGAGUACGCGCGCAAGCGUG<br>AAACUCAAAGGAAUUGACGGGGGCCCGCACAAGCGGUGG<br>AGUAUGUGGUUUAAUUCGAUGCAACGCGAAGAACCUUAC<br>CAGGGCUUGACAUGUCCGGAACCUCAGGGAAACUUGGGG<br>GUGCCUACGGGAACCGGAACACAGGUGGUGCAUGGCUGU<br>CGUCAGCUCGUGUCGUGAGAUGUUGGGUUAAGUCCCGCA<br>ACGAGCGCAACCCUCGUUUUUAGUUGCCAGCAUUAAGUU<br>GGGCACUCUAGAGAGACUGCCGGUGACAAACCGGAGGAA<br>GGUGGGGAUGACGUCAAGUCAGCAUGCCCCUUACGUCCU<br>GGGCUACACACGUACUACAAUGCUACGGACAAAGGGAAG<br>CAAAGCAGCGAUGCCAAGCAAAUCUCAUAAACCGUGGCU<br>CAGUUCAGAUUGCAGGCUGCAACUCGCCUGCAUGAAGGC<br>GGAAUCGCUAGUAAUCGCCGGUCAGCCAUACGGCGGUGA<br>AUACGUUCCCGGGCCUUGUACACACCGCCCGUCACACCA<br>UGGAAGCUGGCUAUGCCCGAAGUCGUUACCCUAACCCUU<br>GUGGAGGGGGAUGCCGAAGGCAGAGCUGGUGACUGGGGU<br>GAAGUCGUAACAAGGUAGCCGUACCGGAAGGUGUGGCUG<br>GAUCACCUCCUUU	16s RNA

Table S3. List of templates selected on SWISS-MODEL for comparative modeling. The first column shows the name of the protein, in the second the template chosen with the corresponding PDB entry; the third column shows the number of residues of the template sequence and the last column shows the organism to which the selected template belongs.

Protein	Organism	Lenght of the sequence template	Template
S1	Spinacia Oleracea	370	30s ribosomal protein S1, chloroplastic PDB *entry:* 5X8P (Ahmed T. et al., 2017) PDB *entity seq:* 5X8P_58
S2	Spinacia Oleracea	236	30s ribosomal protein s2, chloroplastic PDB *entry:* 5X8R (Ahmed T. et al., 2017) PDB *entity seq:* 5X8R_1
S3	Acinetobacter Baumannii	250	30s ribosomal protein S3 PDB *entry*: 6V3E (Morgan et al., 2019) PDB *entity seq:* 6V3E_3
S4	Spinacia Oleracea	201	30s ribosomal protein s4, chloroplastic PDB *entry:* 5X8P (Ahmed T. et al., 2017) PDB *entity seq:* 5X8P_54
S5	Spinacia Oleracea	253	30s ribosomal protein s5, chloroplastic PDB *entry:* 5X8P (Ahmed T. et al., 2017) PDB *entity seq:* 5X8P_38
S6	Spinacia Oleracea	211	Plastid ribosomal protein bS6c, PDB *entry:* 5MMJ (Bieri P. et al., 2017) PDB *entity seq:* 5MMJ_8
S7	Lactococcus lactis subsp. cremoris MG1363	155	30s ribosomal protein s7 PDB *entry:* 5MYJ (Franken et al., 2017) PDB *entity seq:* 5MJY_7
S8	*Staphylococcus Aureus* NCTC 8325	132	30s ribosomal protein s8 PDB *entry:*5ND9 (Khusainov et al, 2017) PDB *entity seq:* 5ND9_8
S9	*Spinacia Oleracea*	197	Ribosomal protein s9 PDB *entry:* 4V61 (Sharma et al., 2007) PDB *entity seq:* 4V61_9
S10	*Lactococcus lactis* subsp. cremoris MG1363	102	30s ribosomal protein s10 PDB *entry:* 5MYJ (Franken et al., 2017) PDB *entity seq:* 5MJY_10

S11	*Staphylococcus Aureus* NCTC 8325	129	30s ribosomal protein s11 PDB *entry:*5ND8 (Khusainov et al, 2017) PDB *entity seq:* 5ND8_11
S12	*Mycolicibacterium Smegmatis* MC2 155	124	30s ribosomal protein s12 PDB *entry:* 5ZEB (Mishra et al., 2018) PDB *entity seq:* 5ZEB_9
S13	*Thermus Thermophilus* HB8	126	30s ribosomal protein s13 PDB *entry:* 3T1H (Murphy et al., 2011) PDB *entity seq:* 3T1H_13
S14	*Acinetobacter Baumannii* AB0057	101	30s ribosomal protein s14 PDB *entry:* 6V39(Morgan et al., 2019) PDB *entity seq:* 6V39_44
S15	*Staphylococcus Aureus* NCTC 8325	89	30s ribosomal protein s15 PDB *entry:*5ND9 (Khusainov et al, 2017) PDB *entity seq:* 5ND9_15
S16	*Spinacia Oleracea*	88	30s ribosomal protein s16, chloroplastic PDB *entry:* 5X8P (Ahmed T. et al., 2017) PDB *entity seq:* 5X8P_45
S17	*Thermus Thermophilus* HB8	105	30s ribosomal protein s17 PDB *entry:* 4V97 (Fagan et al., 2012) PDB *entity seq:* 4V97_17
S18	*Bacillus subtilis* Subsp. subtilis Str. 168	71	30s ribosomal protein s18 PDB *entry:* 5NJT (Beckert et al., 2017) PDB *entity seq:* 5NJT_18
S19	*Spinacia Oleracea*	92	Ribosomal protein s19 PDB *entry:* 4V61 (Sharma et al., 2007) PDB *entity seq:* 4V61_19
S20	*Spinacia Oleracea*	202	Ribosomal protein s20 PDB *entry:* 4V61 (Sharma et al., 2007) PDB *entity seq:* 4V61_20
S21	*Lactococcus lactis* subsp. cremoris MG1363	58	30s ribosomal protein s21 PDB *entry:* 5MYJ (Franken et al., 2017) PDB *entity seq:* 5MJY_21

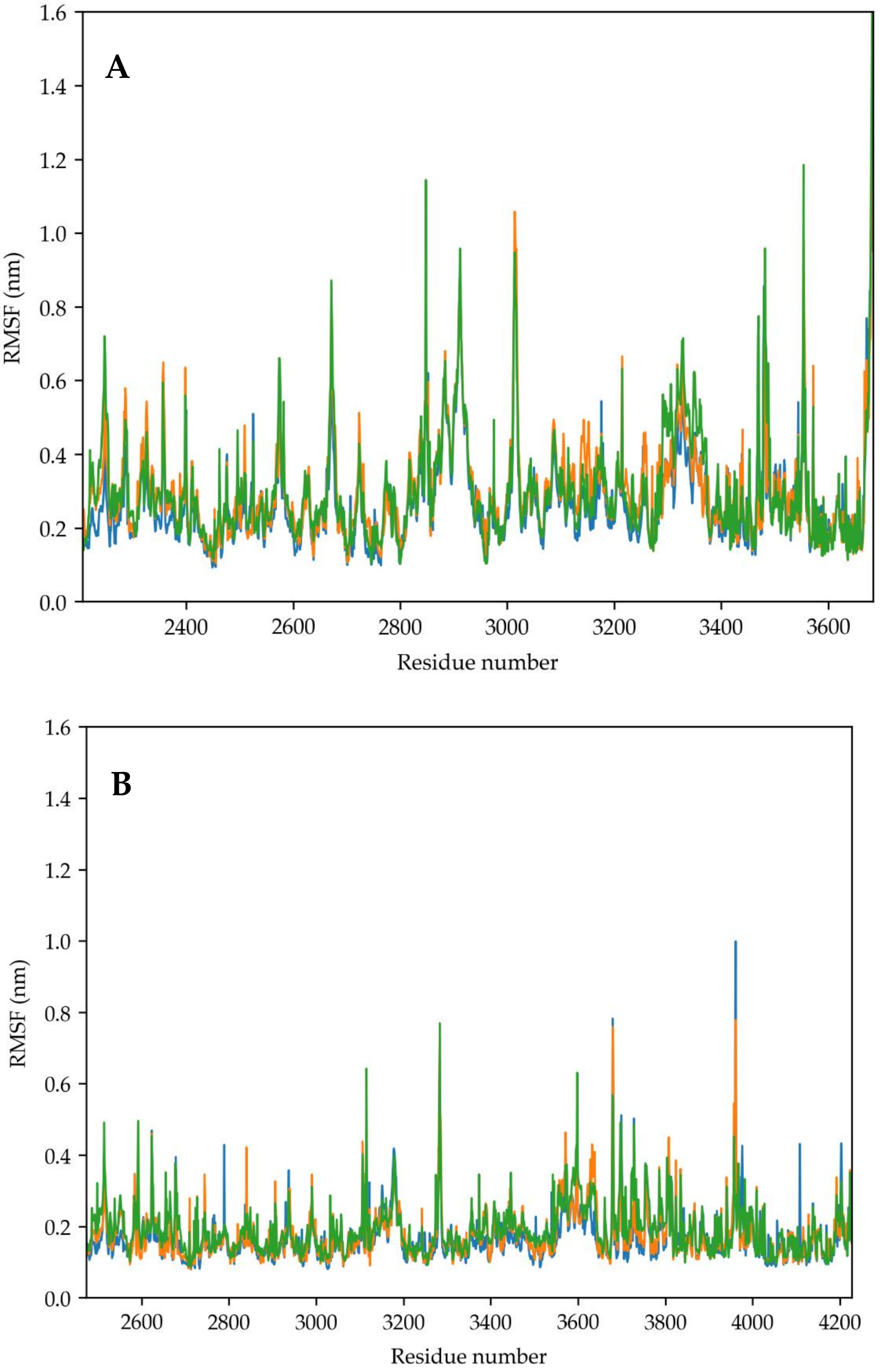

Figure S1. A) RMSF calculated for the C2′ atoms of the RNA in the systems without trehalose and **B)** for the system with trehalose. The blue, orange and green colours identify the three different simulation replicas.

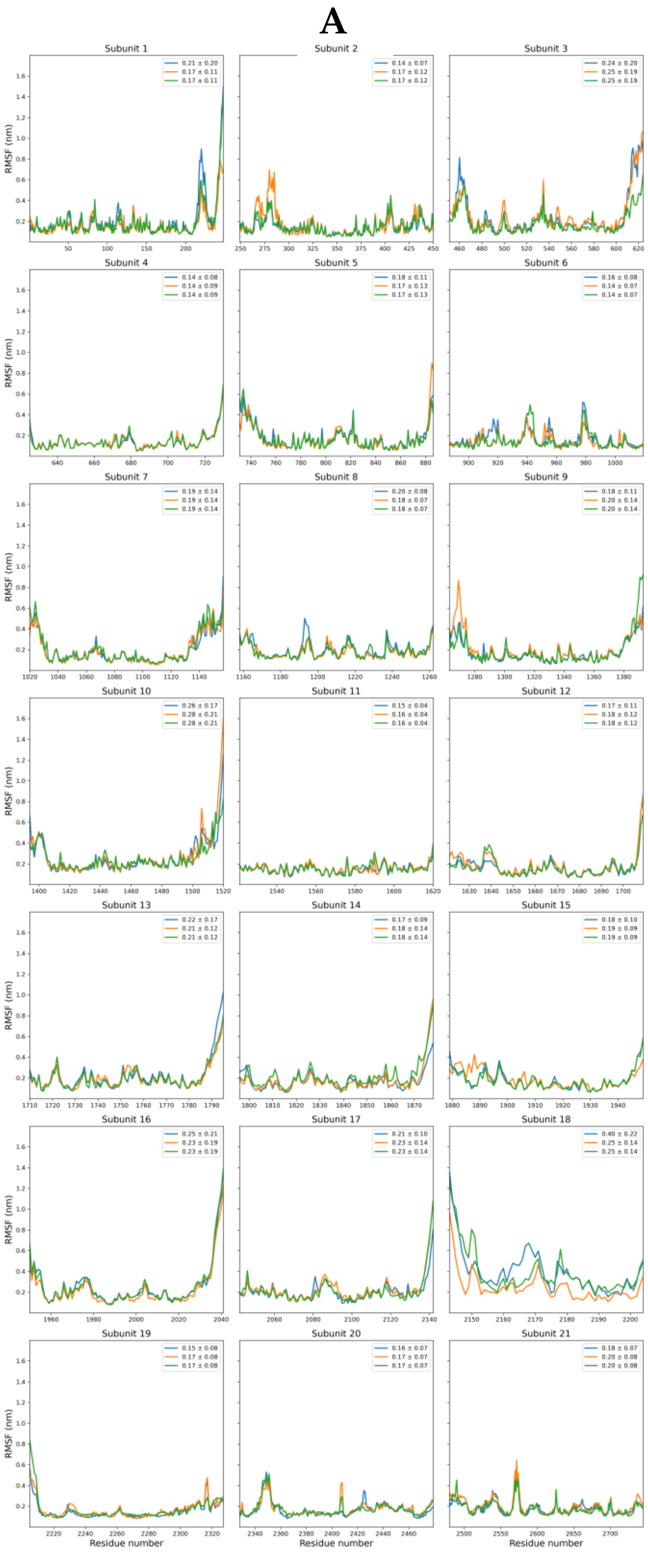
A
Subunit 1
0.21 ± 0.20
0.17 ± 0.11
0.17 ± 0.11
Subunit 2
0.14 ± 0.07
0.17 ± 0.12
0.17 ± 0.12
Subunit 3
0.24 ± 0.20
0.25 ± 0.19
0.25 ± 0.19
Subunit 4
0.14 ± 0.08
0.14 ± 0.09
0.14 ± 0.09
Subunit 5
0.18 ± 0.11
0.17 ± 0.13
0.17 ± 0.13
Subunit 6
0.16 ± 0.08
0.14 ± 0.07
0.14 ± 0.07
Subunit 7
0.19 ± 0.14
0.19 ± 0.14
0.19 ± 0.14
Subunit 8
0.20 ± 0.08
0.18 ± 0.07
0.18 ± 0.07
Subunit 9
0.18 ± 0.11
0.20 ± 0.14
0.20 ± 0.14
Subunit 10
0.26 ± 0.17
0.28 ± 0.21
0.28 ± 0.21
Subunit 11
0.15 ± 0.04
0.16 ± 0.04
0.16 ± 0.04
Subunit 12
0.17 ± 0.11
0.18 ± 0.12
0.18 ± 0.12
Subunit 13
0.22 ± 0.17
0.21 ± 0.12
0.21 ± 0.12
Subunit 14
0.17 ± 0.09
0.18 ± 0.14
0.18 ± 0.14
Subunit 15
0.18 ± 0.10
0.19 ± 0.09
0.19 ± 0.09
Subunit 16
0.25 ± 0.21
0.23 ± 0.19
0.23 ± 0.19
Subunit 17
0.21 ± 0.10
0.23 ± 0.14
0.23 ± 0.14
Subunit 18
0.40 ± 0.22
0.25 ± 0.14
0.25 ± 0.14
Subunit 19
0.15 ± 0.08
0.17 ± 0.08
0.17 ± 0.08
Subunit 20
0.16 ± 0.07
0.17 ± 0.07
0.17 ± 0.07
Subunit 21
0.18 ± 0.07
0.20 ± 0.08
0.20 ± 0.08
RMSF (nm)
Residue number

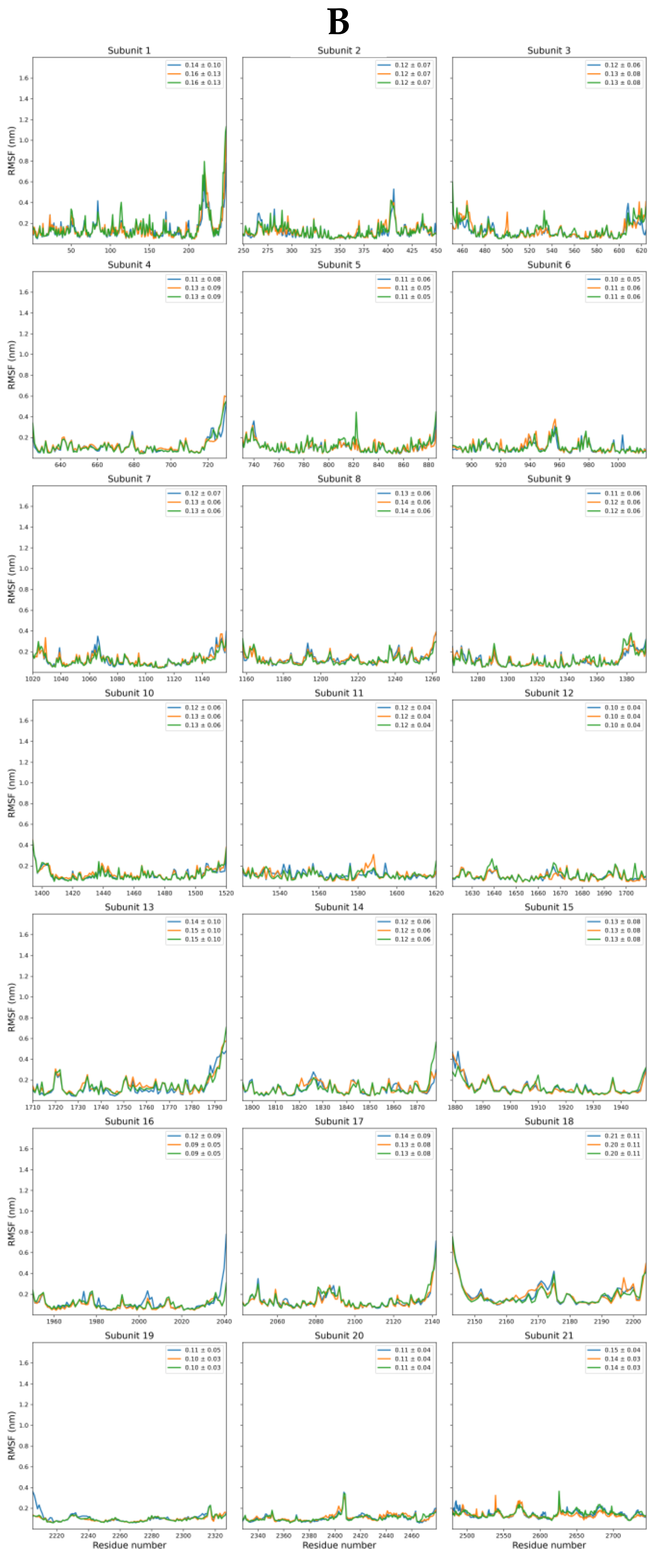

Figure S2. A) RMSF calculated for each of the 21 proteins in the three system replicas simulated without trehalose and **B)** for three system replicas simulated in presence of trehalose). In order, from left to right, the protein chains are shown from S1 to S21. The mean and the standard deviation of each curve is reported inside the plots. The blue, orange and green colours identify the three different simulation replicas.

Table S4. Average percentage of secondary structures for the 21 proteins of 30S ribosomal subunit. For each protein, the first row is related to the system simulated without trehalose, while the second row is related to the system simulated with trehalose.

Protein	Replica	Coil (%)	Helix (%)	Sheet (%)	Turn (%)
**S1 no thr**	1	41.00	27.03	20.00	11.70
	2	41.70	26.50	20.10	11.70
	3	40.90	26.20	20.80	12.20
**S1 thr**	1	41.50	28.70	19.40	10.30
	2	41.20	29.30	19.50	10.10
	3	41.50	28.10	19.40	11.00
**S2 no thr**	1	37.10	35.70	8.90	18.20
	2	38.60	35.60	9.00	16.70
	3	38.20	35.40	8.90	17.50
**S2 thr**	1	39.70	35.80	8.90	15.70
	2	39.20	35.80	8.90	16.20
	3	39.30	35.40	8.90	16.40
**S3 no thr**	1	39.30	23.50	26.80	10.50
	2	36.60	24.10	27.60	11.70
	3	37.70	24.20	26.20	11.90
**S3 thr**	1	40.00	24.10	28.30	7.60
	2	40.50	22.70	27.60	9.20
	3	39.60	22.80	28.70	8.90
**S4 no thr**	1	24.20	27.80	41.70	6.30
	2	23.80	28.00	42.20	6.00
	3	23.80	28.00	41.70	6.50
**S4 thr**	1	22.20	28.20	42.80	6.70
	2	24.50	27.50	42.40	5.60
	3	21.80	28.20	43.70	6.30
**S5 no thr**	1	34.20	54.00	5.10	6.70
	2	31.90	54.00	6.20	7.90
	3	33.60	52.70	6.00	7.80
**S5 thr**	1	30.70	56.80	7.10	5.40
	2	30.60	56.90	7.20	5.30
	3	31.10	57.70	6.50	4.70
**S6 no thr**	1	38.40	25.50	26.20	10.00
	2	36.70	24.30	26.30	12.70
	3	38.40	23.30	26.20	12.00
**S6 thr**	1	36.30	27.40	28.40	7.80
	2	36.70	27.40	28.20	7.60
	3	36.50	27.30	28.20	8.10
**S7 no thr**	1	40.20	31.10	19.10	9.50
	2	40.50	31.00	19.20	9.30
	3	40.30	30.40	19.20	10.10
**S7 thr**	1	40.60	30.30	18.80	10.30
	2	40.20	30.00	18.70	11.00
	3	41.30	28.50	18.80	11.40
**S8 no thr**	1	44.00	20.30	25.60	10.10
	2	41.00	19.90	30.70	8.40
	3	43.80	19.20	27.20	9.70

**S8 thr**	1	47.60	20.00	23.00	9.40
	2	45.60	20.20	25.10	9.00
	3	46.60	20.10	23.50	9.80
**S9 no thr**	1	34.60	26.00	28.60	10.80
	2	35.00	25.20	28.30	11.40
	3	36.10	26.20	28.40	9.30
**S9 thr**	1	34.50	25.50	28.70	11.30
	2	34.80	25.70	28.80	10.80
	3	38.00	25.70	27.40	8.90
**S10 no thr**	1	37.60	46.60	1.60	14.20
	2	37.60	46.00	1.80	14.60
	3	38.00	45.30	1.60	15.00
**S10 thr**	1	38.50	48.10	1.60	11.80
	2	38.60	48.10	1.50	11.80
	3	39.10	48.50	1.60	10.80
**S11 no thr**	1	34.40	55.40	1.10	9.10
	2	33.80	55.60	0.30	10.30
	3	34.60	53.30	1.10	11.00
**S11 thr**	1	32.30	55.80	1.80	10.10
	2	32.60	56.50	1.70	9.20
	3	33.30	55.80	1.40	9.60
**S12 no thr**	1	19.80	72.60	0.10	7.60
	2	18.70	72.90	0.10	8.30
	3	18.90	73.40	0.10	7.60
**S12 thr**	1	16.90	77.00	0.00	6.10
	2	16.40	75.60	0.10	7.90
	3	18.20	76.10	0.00	5.70
**S13 no thr**	1	46.10	23.80	16.90	13.10
	2	49.80	22.60	15.70	11.80
	3	48.60	22.60	16.80	12.10
**S13 thr**	1	49.90	22.90	16.70	10.50
	2	49.00	22.70	16.50	11.70
	3	50.30	22.00	18.00	9.70
**S14 no thr**	1	60.80	0.00	30.10	9.10
	2	57.10	0.60	31.50	10.80
	3	59.90	0.70	28.60	10.90
**S14 thr**	1	57.30	0.00	32.70	9.90
	2	57.00	0.00	33.30	9.70
	3	58.80	0.00	31.60	9.60
**S15 no thr**	1	54.10	35.90	0.00	10.00
	2	51.30	34.80	0.00	14.00
	3	53.50	35.40	0.00	11.10
**S15 thr**	1	53.10	33.30	0.00	13.70
	2	52.50	33.30	0.00	14.30
	3	52.80	32.10	0.00	15.00
**S16 no thr**	1	47.30	23.50	17.20	12.00
	2	47.30	23.00	17.30	12.30
	3	46.50	23.30	17.50	12.70
	1	47.10	26.60	16.50	9.70

**S16 thr**	2	48.70	25.00	17.20	9.10
	3	48.50	25.10	16.90	9.50
**S17 no thr**	1	31.80	54.50	0.70	13.00
	2	34.00	53.80	0.50	11.70
	3	32.40	55.10	0.40	12.20
**S17 thr**	1	26.20	58.80	1.70	13.40
	2	25.30	58.60	1.50	14.50
	3	27.20	58.10	1.60	13.10
**S18 no thr**	1	46.10	39.40	0.20	14.30
	2	47.10	40.60	0.00	12.30
	3	43.70	40.00	0.00	16.30
**S18 thr**	1	39.90	42.70	0.00	17.40
	2	43.00	43.60	0.00	13.40
	3	36.90	47.80	0.00	15.20
**S19 no thr**	1	57.10	6.60	25.50	10.70
	2	57.70	7.00	24.90	10.40
	3	56.10	7.70	26.30	9.90
**S19 thr**	1	56.40	6.10	25.40	12.10
	2	53.60	7.10	26.10	13.20
	3	55.40	6.20	25.80	12.70
**S20 no thr**	1	25.40	34.40	24.90	15.30
	2	25.10	34.40	24.60	15.80
	3	25.10	34.80	23.80	16.30
**S20 thr**	1	26.10	35.80	23.70	14.40
	2	25.90	35.20	23.80	15.10
	3	25.20	36.10	23.80	15.00
**S21 no thr**	1	30.80	43.70	10.0	15.50
	2	31.10	43.80	9.30	15.40
	3	30.60	43.80	9.60	16.00
**S21 thr**	1	30.60	44.70	9.90	14.80
	2	30.40	44.60	10.10	14.90
	3	31.70	43.40	10.00	15.00

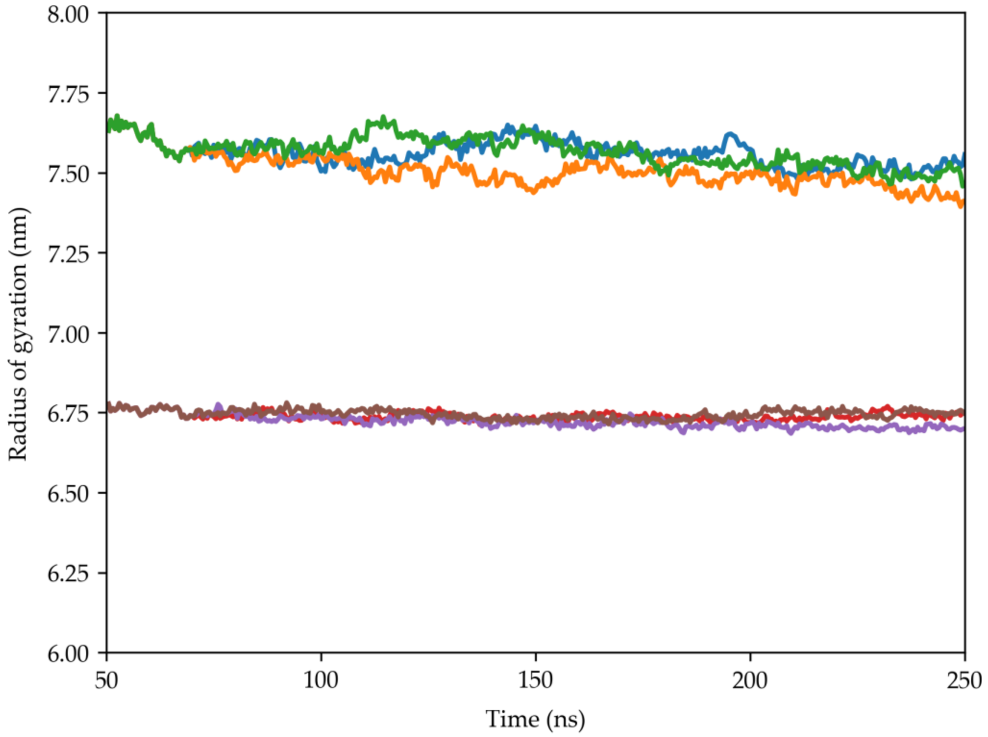

Figure S3. Gyration radius (RG): blue, orange and green lines indicate the RG values calculated for the three replicas in the absence of trehalose, while red, violet and brown lines the values calculated for the replicas simulated in presence of trehalose.
